# Circulating miR-29c, miR-195, and miR-486 Are Objective Indicators to Determine the Moderate Intensity of Resistance Exercise

**DOI:** 10.7759/cureus.76212

**Published:** 2024-12-22

**Authors:** Daisuke Takamura, Kentaro Iwata, Shota Inoue, Junpei Hatakeyama, Hideki Moriyama

**Affiliations:** 1 Department of Rehabilitation, Kobe City Medical Center General Hospital, Kobe, JPN; 2 Department of Rehabilitation Science, Graduate School of Health Science, Kobe University, Kobe, JPN; 3 Life and Medical Sciences Area, Health Sciences Discipline, Kobe University, Kobe, JPN

**Keywords:** aerobic exercise, exercise intensity, micrornas, moderate exercise, resistance exercise

## Abstract

Background and objective

Moderate exercise is important for health; however, there are variations among individuals in terms of characterizing moderate intensity and it is difficult to identify. In light of this, the purpose of this study was to identify new objective indicators to determine effective exercise intensity.

Methods

This study involved both human and animal experiments. After subjecting mice to exercise of effective intensity, microarray analysis of circulating microRNA expression was conducted to identify candidates for objective indicators to determine effective exercise intensity. Then, we assessed if these microRNAs were altered after aerobic or resistance exercises in humans using quantitative real-time PCR. Twelve healthy males were randomly assigned to two groups: the low-intensity exercise group (LI group) and the high-intensity exercise group (HI group) and they underwent four weeks of exercise program.

Results

Based on microarray analysis, 188 microRNAs were altered after aerobic exercise, and 167 microRNAs were altered after resistance exercise. Combining these findings with the data from some published reports, we selected miR-29c, miR-23b, miR-222, miR-195, miR-126, miR-133a, and miR-486 as the candidates for biomarkers to determine the effective exercise intensity. In the human study, physical performance improved after resistance exercise only in the HI group. Of the microRNAs, miR-29c, miR-195, and miR-486 increased immediately after resistance exercise only in the HI group. Fold change of miR-486 correlated with changes in knee extensor strength (r=0.744, *p*=0.005).

Conclusions

Resistance exercise at effective intensity upregulated the expression of miR-29c, miR-195, and miR-486. Hence, these microRNAs may serve as objective indicators to determine the intensity of resistance exercise. Among them, miR-486 may aid in predicting the response to resistance exercise.

## Introduction

Regular exercise is crucial for preventing and managing various diseases, including lifestyle diseases. According to worldwide statistics [1], 27.5% of adults did not meet the 2010 global recommendations on physical activity for health prescribed by the World Health Organization (WHO). If the global population becomes more physically active, four to five million deaths may be averted annually. The WHO recommendations have highlighted the importance of exercise. Exercise is also one of the most effective treatments in rehabilitation medicine. The intensity of the exercise is crucial: suboptimal levels fail to yield benefits, yet excessive intensity can also lead to adverse effects. Accordingly, achieving the maximum benefit requires ensuring a “moderate” level of exercise.

Exercise is broadly divided into aerobic and resistance exercise. Aerobic exercise focuses on pumping the oxygenated blood from the heart to other working skeletal muscles and has beneficial effects on cardiorespiratory fitness. Maximum oxygen uptake (VO_2max_) or maximum heart rate is often used to determine the exercise intensity. Guidelines from the American College of Sports Medicine and the American Heart Association [2] recommend medium- to vigorous-intensity exercises several days a week. Aerobic exercise is typically prescribed at 50-85% of maximum intensity in clinical practice. However, dedicated equipment is required for the measurement of VO_2max_, and the measurement process is complicated and time-consuming. Heart rate is inadequate as an objective indicator to determine exercise intensity since it is affected by inflammation and other general conditions. Resistance exercise focuses on resisting self-weight or external resistance and is effective for improving muscle strength.

Maximal voluntary contraction (MVC) or repetition maximum (RM) are recommended to determine the exercise intensity. Per the overload principle, medium to vigorous intensity over 60% MVC or 70% 1RM is endorsed to increase the effects of resistance exercise. Resistance exercise with higher intensity leads to muscle hypertrophy and muscle mass gain [3]. However, the maximum effort must be imposed on the subject in advance for using the MVC or RM methods. Therefore, these methods are difficult to apply to frail elderly or patients with cardiovascular disease. Also, weight machines are not always available in clinical practice or in-home exercise settings, which limits resistance exercise using MVC or RM methods. The use of ratings of perceived exertion is a convenient and practical method in both aerobic and resistance exercise. One of the ratings of perceived exertion is the Borg scale. Nevertheless, there are sex- and age-related differences in perceived exertion [4]. Additionally, the Borg scale is also difficult to apply to subjects with impaired cognitive function, which limits the exercise with ratings of perceived exertion. Hence, new objective indicators are needed as an alternative to existing indicators to prescribe effective exercise.

MicroRNAs are a type of small, noncoding RNA that regulate post-transcriptional gene expression by binding to target messenger RNAs, leading to messenger RNA degradation or translation repression [5]. MicroRNAs can be detected in the circulation and are remarkably stable in body fluids, since microRNAs are packaged in microparticles, including exosomes, microvesicles, and apoptotic bodies [5]. Consequently, their utility as new biomarkers has been reported in various studies. Blood possesses the most available information and provides the most stable results among body fluids; therefore, circulating microRNAs in the blood are reasonably well-suited to be used as biomarkers. Among over 1000 microRNAs, some are altered after exercise [5], such as aerobic and resistance exercises. Moreover, different exercise volumes may result in different microRNA expressions after aerobic and resistance exercises [6,7]. In light of these data and findings, we speculate that circulating microRNAs in the blood can be used to determine exercise intensity. While microRNAs being altered by just exercise has been reported in studies, the specific effects of moderate exercise intensity on microRNAs are unknown.

Although medium- to vigorous-intensity exercise has been recommended to have beneficial effects in humans [2,3], individuals differ in terms of optimal exercise intensity, which is difficult to identify. In this study, we comprehensively explored altered microRNAs after mice were subjected to exercise at medium to vigorous intensity, which served as candidates for indicators to determine moderate exercise intensity. Subsequently, the utility of these microRNAs was validated in humans. The purpose of this study was to identify new objective indicators to determine effective exercise intensity.

This article was previously posted to the Research Square preprint server on October 18, 2024.

## Materials and methods

Animal experiments

The experimental procedures were approved by the Kobe University Animal Care and Use Committee (approval number: P190814) in August 2019. Nine male C57BL/6 mice aged eight weeks were used as the research sample from Japan SLC Inc. The mice were housed in polycarbonate cages with bedding and were maintained under artificial conditions at a constant temperature of 22 ± 1 °C with a constant humidity of 55 ± 5% and a 12-hour dark/12-hour light cycle. They had free access to water and food 24 hours a day. All animals were fasted overnight before sacrifice and euthanized under general anesthesia and analgesia at the end of the experimental period.

The mice were randomly divided into three groups: the normal caged mouse group (the control group, n=3); the aerobic exercise group (AE group, n=3), and the resistance exercise group (RE group, n=3). The intensity of each exercise protocol is described below. These exercise protocols have been previously carried out in our laboratory with no adverse events.

Animal Exercise Protocol

In the AE group, acute exercise was conducted using a treadmill (MK-680, Muromachi Kikai Co., Ltd., Tokyo, Japan) at 20 m/min for 60 minutes with 0° incline. Before and after the exercise, a five-minute warm-up and cool-down was conducted at 8 m/min. The mice were trained to be accustomed to aerobic exercise using a treadmill one week before the acute exercise. The detailed procedures are presented in Table 1.

**Table 1:** The ptotocols of animal exercise AE group: aerobic exercise group; RE group: resistance exercise group

Day	Control group	AE group	RE group
Day 1	Normal cage	0 m/min for 30 minutes	Housed in a chamber for 15 minutes
Day 2		0 m/min for 30 minutes and 8 m/min for 15 minutes	4 sets of 3 climbing exercises without resistance weight
Day 3		0 m/min for 15 minutes and 8 m/min for 30 minutes	Normal cage
Day 4		0 m/min for 15 minutes and 8 m/min for 45 minutes	Normal cage
Day 5		8 m/min for 60 minutes	4 sets of 3 climbing exercises without resistance weight
Day 6		Normal cage	Normal cage
Day 7		Normal cage	Normal cage
Day 8		20 m/min for 60 minutes followed by blood sampling	4 sets of 3 climbing exercises with a weight (120 % of body weight) followed by blood sampling

In the RE group, acute exercise was conducted using a ladder (110 cm high, 10 cm wide, 1 cm space between steps). The ladder was placed at an 80° incline, and a resting chamber (13 cm, 10 cm, 30 cm) was placed at the top of the ladder. The mice climbed the ladder carrying a weight equivalent to 120% of its body weight attached to the base of the tail. The resistance exercise consisted of four sets of three climbing exercises from the base to the top of the ladder, interspaced with a minute between each climbing and with two minutes between each set. The mice were familiarized with the resistance exercise apparatus by making them climb the ladder without resistance weight one week before the acute exercise. The detailed procedures are shown in Table 1.

Animal Blood Sampling

About 0.5 ml of blood samples were taken from the inferior vena cava of each mouse three hours after each acute exercise and then centrifuged at 3,000 × g for 10 minutes at 4 ℃ to separate plasma. Then the plasma was centrifuged at 16,000 × g for 10 minutes at 4 ℃ to remove cell debris, and 100 μl of plasma was collected. For microarray analysis, the samples of three mice from the same group were mixed to obtain enough plasma. Finally, 300 μl of plasma was obtained from each group.

Microarray Analysis of microRNA Expression

Total RNA was extracted from 300 μl plasma samples using a 3D-Gene RNA extraction reagent supplied with a liquid sample kit (Toray Industries, Inc., Kanagawa, Japan). Comprehensive microRNA expression analysis was conducted using a 3D-Gene miRNA Labeling kit and 3D-gene Human miRNA Oligo Chip (Toray Industries, Inc., Kanagawa, Japan), which was designed to detect 2588 microRNA sequences registered in the miRBase release 21 (https://www.mirbase.org/). The data from this analysis were entered into the Gene Expression Omnibus (GEO) database under accession number (GSE283677). MicroRNAs with signal intensity |log_2_ Fold change| > 1 in comparison with the control group were considered detected microRNAs.

Target genes of detected microRNAs were investigated using miRWalk (http://mirwalk.umm.uni-heidelberg.de/). The predicted target genes were used for further pathway analysis. The online tool database for annotation, visualization, and integrated discovery (DAVID), version 6.8 (https://david.ncifcrf.gov/home.jsp) was used to conduct gene ontology (GO) analysis and pathway analysis. Kyoto Encyclopedia of Genes and Genomes (KEGG) pathway with a false discovery rate (FDR) <0.05 was selected.

Human experimental design

The study was conducted with the approval of the Ethics Committee of Kobe City Medical Center General Hospital (n201201) in December 2020. Participants were recruited at our hospital between January 2021 and December 2021. Subsequent biochemical experiments and statistical analyses were completed in December 2022. Blood samples were collected according to the protocols of the Ethics Committee of our hospital. Written informed consent was obtained from all participants before enrolment. The human study comprised two experiments: an aerobic exercise study and a resistance exercise study. Expressions of microRNAs are influenced by various diseases and sex hormones; therefore, 12 healthy males were recruited for each study. They were free of any diseases or injuries that would impair their physical performance and had no habit of smoking. None of the participants exercised regularly.

**Figure 1:**
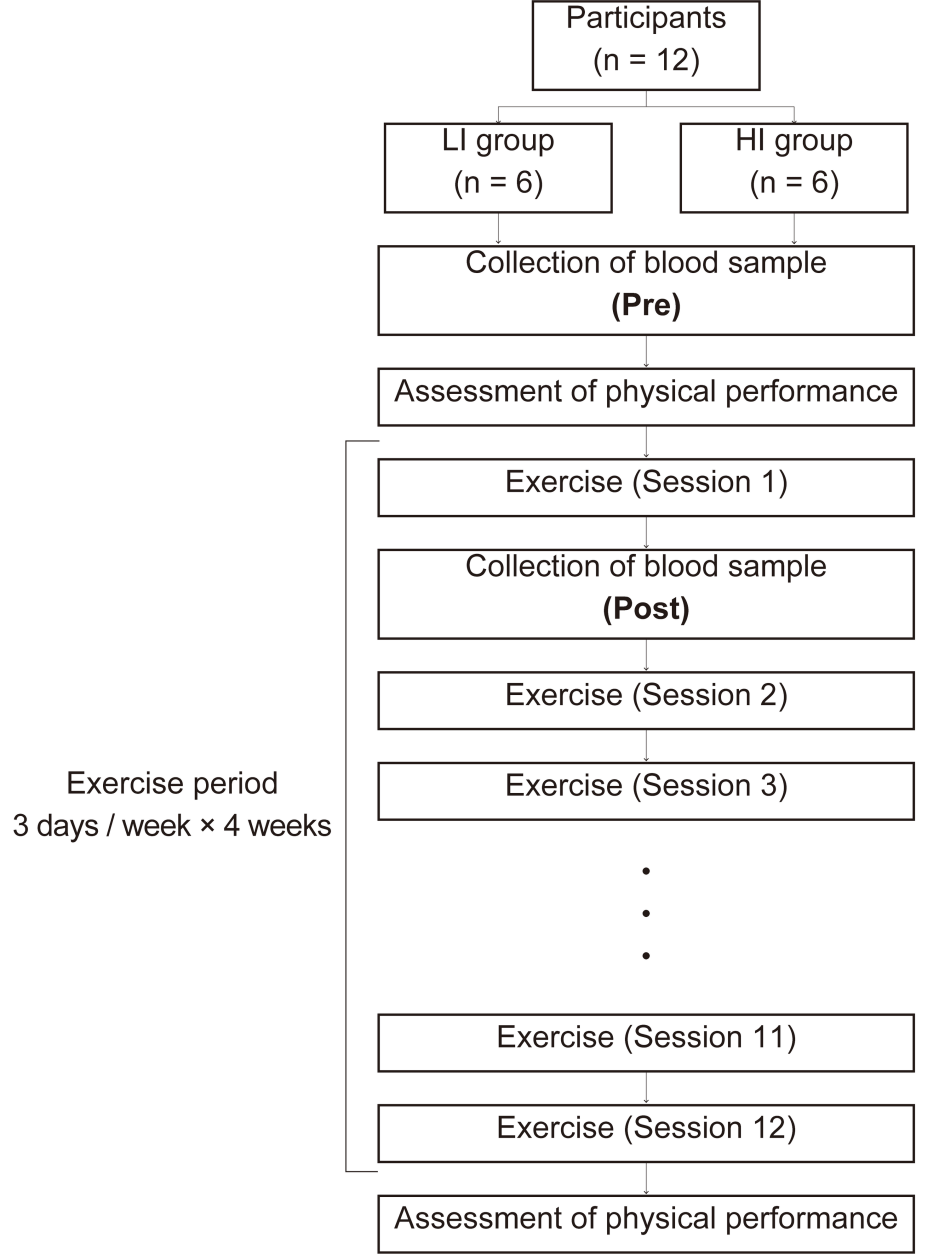
Flow chart depicting the human study

The participants were randomly divided into two groups of six males each using a random number table: low-intensity exercise group (LI group) and high-intensity exercise group (HI group). Each participant undertook a supervised three-day/week aerobic exercise or resistance exercise program for four weeks (total of 12 sessions). The details of exercise programs and intensity for each group in aerobic and resistance exercise are described below. Before and after four weeks of exercise period, physical performance was assessed. For each group, a blood sample was collected before (Pre) and immediately after the first session of the exercise (Post). The blood sample at pre was collected before the physical performance assessment to avoid the acute effect of exercise on circulating microRNAs. Figure 1 provides an overview of the human study.

Aerobic Exercise

As the main outcome of aerobic exercise, VO_2max_ was measured. Before and after four weeks of the aerobic exercise program, the participants underwent cardiopulmonary exercise testing to determine VO_2max_ using a cycle ergometer (Aerobike 75XLⅡ, Combi Wellness, Japan) with portable metabolic system (Mobile Aeromonitor AE-100i, MINATO medical science Co., Ltd, Japan). The workload was gradually increased by 20 watts every minute until oxygen consumption following a minute of unloaded pedaling could not be increased further. Objective criteria for maximal effort included at least two of the following factors: (1) increased workload without a corresponding increase in VO_2_; (2) respiratory exchange quotient equal to or greater than 1.10; and (3) a pedal cadence lower than 50 rpm despite maximal voluntary effort.

Exercise programs were started the week after the baseline physical performance assessment. The participants exercised for 20 minutes three times per week for four weeks using a cycle ergometer (Medergo EM-400, OG Wellness, Japan). The intensity of the exercise program in each group is summarized below; in the LI group, the participants rode at 20% of their VO_2max_. In the HI group, they rode at 70-80% of their VO_2max_. The load was increased gradually to reach the target load in the first three minutes.

Resistance Exercise

As the main outcome for resistance exercise, maximal isometric muscle strength was measured. Maximal isometric knee extensor strength and knee flexor strength were measured on the right leg with a dynamometer (Isoforce GT-330, OG Wellness, Japan) according to the manufacturer’s instructions before and after four weeks of the resistance exercise program. The strength was measured twice and the maximal value was used for the analysis.

Before the 1RM determination, participants were required to rest in a seated position. After a one-minute rest, 1RM for the leg extension and leg curl was measured using the training machine (HUR, Inter Reha, Japan). The load was increased until the participants were unable to lift the load. The participants rested for a few minutes between each trial to avoid fatigue. About three to five trials were conducted to determine 1RM.

Exercise programs were started the week after the baseline physical assessment. Before the exercise, the participants stretched for several minutes. Then they performed warm-up sets of 10 repetitions with no load. The resistance exercise consisted of two consecutive exercises (leg extension and leg curl), comprising five sets of 10 repetitions using the training machine (HUR, Inter Reha, Japan). The intensity of the exercise program in each group is summarized below. In the LI group, the intensity was set at 10% 1RM. In the HI group, the intensity was set at 80% 1RM. The exercises were carried out within two minutes of rest between each set and exercise.

Human Blood Samples

About 2 ml of venous blood samples were obtained from the participant’s forearm at Pre and Post by using EDTA-2K-containing tubes and then centrifuged at 1,900 × g for 10 minutes at 4 ℃ to separate plasma within one hour after collection. Then, the plasma was centrifuged at 16,000 × g for 10 minutes at 4 ℃ to remove cell debris. Plasma in the supernatant was stored at -80 ℃ until further analysis.

Quantification of Circulating microRNAs

Total RNA was isolated from the plasma sample using the mirVana Paris kit (Thermo Fisher Scientific, Waltham, MA) according to the manufacturer’s protocol. Briefly, 300 μl of plasma was used and mixed with a total volume of 2X denaturing solution to inactive RNase. To allow for normalization of sample-to-sample variation in RNA isolation, synthetic C. elegans cel-miR-39-3p (UCACCGGGUGUAAAUCAGCUUG) was added (1 μmol of oligonucleotides in a 3 μmol total volume) as spike-in control. RNA was eluted with 70 μl of elution solution.

TaqManTM MicroRNA assays (Thermo Fisher Scientific) specific for miR-29c-5p (001818), miR-23b-5p (002126), miiR-222-5p (002097), miR-195a-5p (000494), miR-126-5p (000451), miR-133a-3p (002246), miR-486-5p (001278), and miR-39-3p (002000) were used to analyze circulating microRNAs, according to the manufacturer’s instructions. Reverse transcription was carried out with TaqMan MicroRNA Reverse Transcription Kit (Thermo Fisher Scientific) and reverse transcription primers for each microRNA to synthesize cDNA.

Quantitative real-time PCR was carried out using the Applied Biosystems StepOne Real-Time PCR (Applied Biosystems, Foster City, CA) with the cDNA samples, Fast Advanced Master Mix (Thermo Fisher Scientific), and TaqMan microRNA assays. Data were normalized using miR-39 and relative microRNA levels were expressed using the ΔΔCt method. Briefly, the results of real-time PCR were first presented as cycle threshold (Ct) values, and so we calculated the ΔΔCt value for each microRNA using the Ct value of miR-39 at Pre. Using this method, we calculated the fold change for Post.

Statistical analysis

We first evaluated if the variables were normally distributed using the Shapiro-Wilk test. Normally distributed variables were expressed as mean [standard deviation (SD)], and non-normally distributed variables as median [interquartile range(IQR)]. To compare physical performance or microRNA expressions between two time points, paired t-test or Wilcoxon signed-rank test were used. Chi-squared test, t-test, or Mann-Whitney U test were used to compare participants’ characteristics and physical performance. The correlation between microRNAs and changes in physical performance was evaluated using Pearson’s correlation coefficients or Spearman’s rank correlation coefficients.

Data were analyzed using EZR (Saitama Medical Center, Jichi Medical University, Saitama, Japan; http://www.jichi.ac.jp/saitama-sct/SaitamaHP.files/statmedEN.html), which is a graphical user interface for R version 4.2.2 (The R Foundation for Statistical Computing, Vienna, Austria). More precisely, it is a modified version of R commander designed to add statistical functions frequently used in biostatistics. The level of significance was set at *p*<0.05.

## Results

Microarray analysis after exercise

**Figure 2:**
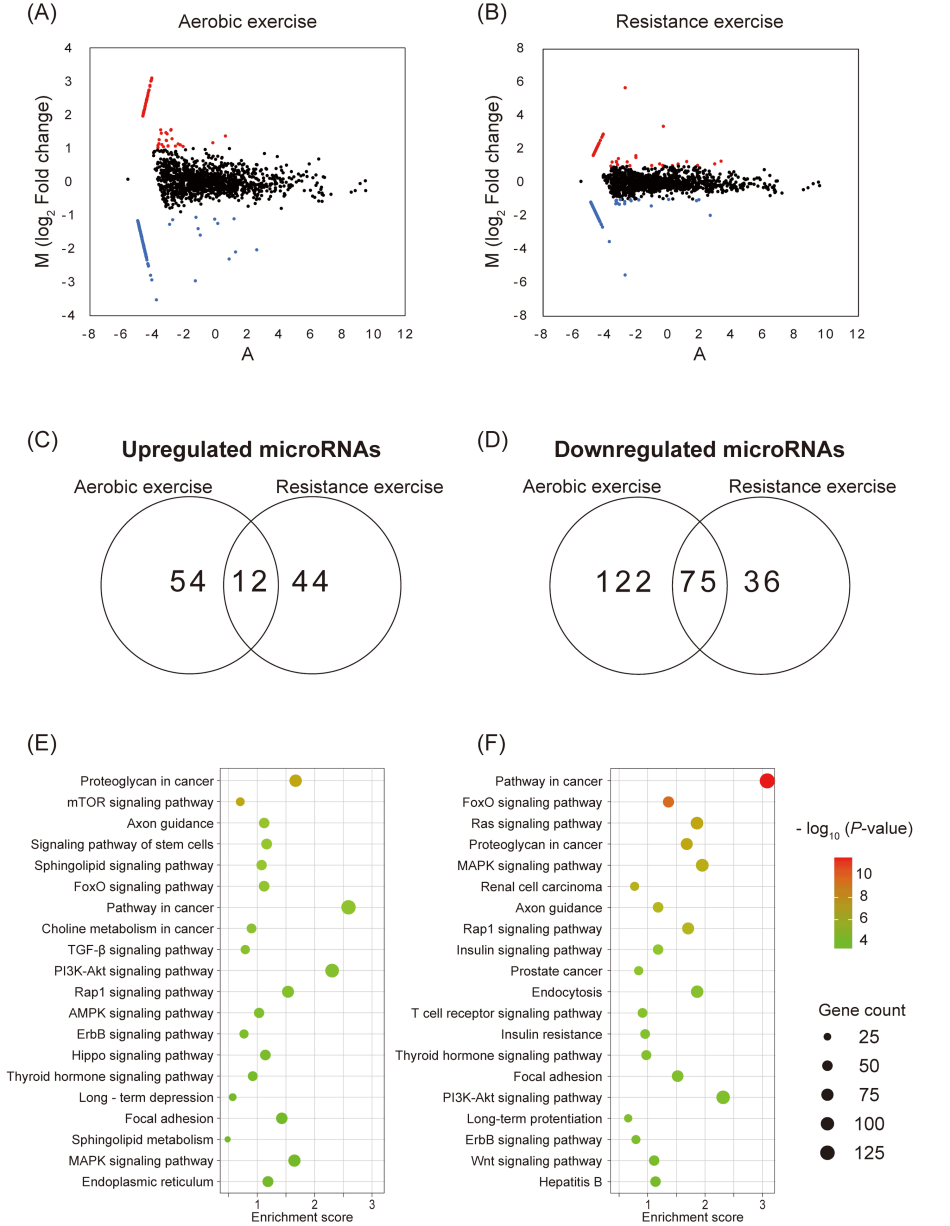
Microarray analysis (A-B) Numbers of upregulated and downregulated miRNAs in the AE group (A) and RE group (B). MicroRNAs were selected using the |log_2_ Fold change| > 1 in comparison with the control group. The red dots represent upregulated microRNAs and the blue dots represent downregulated microRNAs. (C-D) The Venn diagram shows the numbers of upregulated and downregulated microRNAs in the AE group and RE group. (E-F) Bubble charts for gene ontology and KEGG pathway enrichment analysis. The top 20 KEGG enrichment results in the AE group (E) and RE group (F) AE group: aerobic exercise group; KEGG: Kyoto Encyclopedia of Genes and Genomes; RE group: resistance exercise group

Based on microarray analysis, 188 microRNAs (66 upregulated and 122 downregulated) were altered in the AE group, and 167 (56 upregulated and 111 downregulated) were altered in the RE group. Of these, 96 microRNAs (12 upregulated and 75 downregulated) were changed in both the AE group and RE group (|log_2_ Fold change| > 1) (Figures 2A-2D). Tables 2-5 show the altered microRNAs in the AE and RE groups.

**Table 2:** Upregulated microRNAs in the AE group AE group: aerobic exercise group

Rank	microRNAs	Log_2_ fold change	Ratio
1	mmu-miR-6350	3.11	8.60
2	mmu-miR-195b	3.06	8.34
3	mmu-miR-148b-3p	3.02	8.12
4	mmu-miR-465d-3p	3.01	8.05
5	mmu-miR-1957b	2.89	7.41
6	mmu-miR-1928	2.86	7.27
7	mmu-miR-107-5p	2.75	6.72
8	mmu-miR-3618-5p	2.72	6.57
9	mmu-miR-8094	2.66	6.31
10	mmu-miR-450a-5p	2.65	6.29
11	mmu-miR-338-5p	2.59	6.03
12	mmu-miR-383-3p	2.59	6.01
13	mmu-miR-302a-5p	2.55	5.86
14	mmu-miR-666-3p	2.49	5.61
15	mmu-miR-377-3p	2.47	5.53
16	mmu-miR-145a-3p	2.46	5.51
17	mmu-miR-30b-3p	2.46	5.51
18	mmu-miR-7223-3p	2.46	5.50
19	mmu-miR-29c-5p	2.44	5.43
20	mmu-miR-28c	2.42	5.35
21	mmu-miR-7061-3p	2.39	5.25
22	mmu-miR-698-3p	2.39	5.25
23	mmu-miR-1953	2.35	5.09
24	mmu-miR-298-3p	2.28	4.85
25	mmu-miR-6414	2.27	4.81
26	mmu-miR-23b-5p	2.26	4.80
27	mmu-miR-1970	2.26	4.78
28	mmu-miR-3066-5p	2.20	4.59
29	mmu-miR-539-5p	2.16	4.46
30	mmu-miR-669g	2.13	4.38
31	mmu-miR-6335	2.11	4.33
32	mmu-miR-6998-3p	2.11	4.31
33	mmu-miR-5101	2.10	4.29
34	mmu-miR-6907-3p	2.09	4.27
35	mmu-miR-1949	2.09	4.26
36	mmu-miR-7214-5p	2.07	4.20
37	mmu-miR-344c-3p	2.05	4.14
38	mmu-miR-182-3p	2.03	4.10
39	mmu-miR-27b-5p	2.02	4.07
40	mmu-miR-7229-3p	2.01	4.04
41	mmu-miR-135b-5p	2.01	4.04
42	mmu-miR-704	1.98	3.94
43	mmu-miR-6951-3p	1.97	3.91
44	mmu-miR-34a-3p	1.57	2.97
45	mmu-miR-491-3p	1.55	2.93
46	mmu-miR-672-5p	1.55	2.92
47	mmu-miR-219a-2-3p	1.48	2.78
48	mmu-miR-3062-5p	1.46	2.75
49	mmu-miR-381-5p	1.43	2.70
50	mmu-miR-212-3p	1.37	2.58
51	mmu-miR-142a-3p	1.29	2.44
52	mmu-miR-33-3p	1.27	2.41
53	mmu-miR-6401	1.25	2.38
54	mmu-miR-6957-3p	1.23	2.35
55	mmu-miR-1982-5p	1.17	2.25
56	mmu-miR-7657-3p	1.15	2.21
57	mmu-miR-183-3p	1.13	2.20
58	mmu-miR-98-3p	1.11	2.16
59	mmu-miR-466c-5p	1.11	2.15
60	mmu-miR-7a-1-3p	1.09	2.13
61	mmu-miR-1298-3p	1.08	2.11
62	mmu-miR-7043-5p	1.07	2.10
63	mmu-miR-670-5p	1.07	2.10
64	mmu-miR-5626-5p	1.07	2.09
65	mmu-miR-6949-5p	1.05	2.07
66	mmu-miR-325-3p	1.02	2.03

**Table 3:** Downregulated microRNAs in the AE group AE group: aerobic exercise group

Rank	microRNAs	Log_2_ fold change	Ratio
1	mmu-miR-6920-5p	-3.52	0.09
2	mmu-miR-1a-3p	-2.95	0.13
3	mmu-miR-206-3p	-2.92	0.13
4	mmu-miR-1197-5p	-2.78	0.15
5	mmu-miR-802-3p	-2.51	0.18
6	mmu-miR-743b-5p	-2.50	0.18
7	mmu-miR-1843a-3p	-2.46	0.18
8	mmu-miR-28a-5p	-2.44	0.18
9	mmu-miR-743a-5p	-2.43	0.19
10	mmu-miR-6380	-2.33	0.20
11	mmu-miR-133a-3p	-2.31	0.20
12	mmu-miR-339-3p	-2.27	0.21
13	mmu-let-7a-2-3p	-2.27	0.21
14	mmu-miR-302d-5p	-2.26	0.21
15	mmu-miR-5616-5p	-2.24	0.21
16	mmu-miR-1963	-2.23	0.21
17	mmu-miR-496a-5p	-2.22	0.22
18	mmu-miR-129-2-3p	-2.20	0.22
19	mmu-miR-7656-5p	-2.19	0.22
20	mmu-miR-340-3p	-2.18	0.22
21	mmu-miR-7660-5p	-2.18	0.22
22	mmu-miR-7064-3p	-2.16	0.22
23	mmu-miR-329-5p	-2.15	0.23
24	mmu-miR-467e-3p	-2.12	0.23
25	mmu-miR-881-5p	-2.10	0.23
26	mmu-miR-133b-3p	-2.09	0.24
27	mmu-miR-654-3p	-2.08	0.24
28	mmu-miR-7073-5p	-2.07	0.24
29	mmu-miR-701-5p	-2.05	0.24
30	mmu-miR-1948-3p	-2.04	0.24
31	mmu-miR-6912-5p	-2.03	0.25
32	mmu-miR-7216-3p	-2.02	0.25
33	mmu-miR-3063-3p	-2.01	0.25
34	mmu-miR-6372	-2.00	0.25
35	mmu-miR-433-3p	-1.99	0.25
36	mmu-miR-7a-5p	-1.98	0.25
37	mmu-miR-434-5p	-1.97	0.26
38	mmu-miR-302c-5p	-1.96	0.26
39	mmu-miR-691	-1.96	0.26
40	mmu-miR-202-3p	-1.95	0.26
41	mmu-miR-6417	-1.95	0.26
42	mmu-miR-1938	-1.94	0.26
43	mmu-miR-1968-5p	-1.93	0.26
44	mmu-miR-6946-5p	-1.92	0.26
45	mmu-miR-344-5p	-1.92	0.26
46	mmu-miR-382-5p	-1.92	0.26
47	mmu-miR-1668	-1.91	0.27
48	mmu-miR-1955-3p	-1.90	0.27
49	mmu-miR-3544-5p	-1.90	0.27
50	mmu-miR-18b-5p	-1.89	0.27
51	mmu-miR-1903	-1.87	0.27
52	mmu-miR-201-3p	-1.87	0.27
53	mmu-miR-153-5p	-1.86	0.27
54	mmu-miR-3092-5p	-1.85	0.28
55	mmu-miR-708-5p	-1.85	0.28
56	mmu-miR-3106-5p	-1.85	0.28
57	mmu-miR-7077-3p	-1.84	0.28
58	mmu-miR-293-3p	-1.82	0.28
59	mmu-miR-7688-5p	-1.82	0.28
60	mmu-miR-301b-5p	-1.81	0.28
61	mmu-miR-132-3p	-1.80	0.29
62	mmu-miR-196a-1-3p	-1.79	0.29
63	mmu-miR-28a-3p	-1.78	0.29
64	mmu-miR-3063-5p	-1.77	0.29
65	mmu-miR-490-5p	-1.74	0.30
66	mmu-miR-3069-3p	-1.73	0.30
67	mmu-miR-3087-3p	-1.71	0.31
68	mmu-miR-6958-3p	-1.70	0.31
69	mmu-miR-7116-3p	-1.69	0.31
70	mmu-miR-7689-5p	-1.67	0.31
71	mmu-let-7e-3p	-1.66	0.32
72	mmu-miR-713	-1.65	0.32
73	mmu-miR-222-5p	-1.64	0.32
74	mmu-miR-6357	-1.63	0.32
75	mmu-miR-100-3p	-1.62	0.32
76	mmu-miR-1957a	-1.62	0.33
77	mmu-miR-3058-5p	-1.61	0.33
78	mmu-miR-5626-3p	-1.60	0.33
79	mmu-miR-6339	-1.60	0.33
80	mmu-miR-24-1-5p	-1.59	0.33
81	mmu-miR-92a-1-5p	-1.59	0.33
82	mmu-miR-5123	-1.59	0.33
83	mmu-miR-3086-5p	-1.58	0.33
84	mmu-miR-3094-3p	-1.57	0.34
85	mmu-miR-411-5p	-1.56	0.34
86	mmu-miR-871-3p	-1.54	0.34
87	mmu-miR-6407	-1.53	0.35
88	mmu-miR-871-5p	-1.53	0.35
89	mmu-miR-882	-1.48	0.36
90	mmu-miR-598-3p	-1.47	0.36
91	mmu-miR-6969-3p	-1.46	0.36
92	mmu-miR-217-5p	-1.45	0.37
93	mmu-miR-6342	-1.44	0.37
94	mmu-miR-6374	-1.43	0.37
95	mmu-miR-7086-3p	-1.43	0.37
96	mmu-miR-466n-5p	-1.41	0.38
97	mmu-miR-669b-5p	-1.40	0.38
98	mmu-miR-5125	-1.39	0.38
99	mmu-miR-707	-1.38	0.38
100	mmu-miR-377-5p	-1.38	0.39
101	mmu-miR-1897-3p	-1.37	0.39
102	mmu-miR-3080-5p	-1.35	0.39
103	mmu-miR-181c-5p	-1.35	0.39
104	mmu-miR-7675-3p	-1.33	0.40
105	mmu-miR-1912-5p	-1.32	0.40
106	mmu-miR-7660-3p	-1.31	0.40
107	mmu-miR-6898-3p	-1.30	0.41
108	mmu-miR-541-5p	-1.29	0.41
109	mmu-miR-7018-3p	-1.27	0.42
110	mmu-miR-379-5p	-1.27	0.42
111	mmu-miR-467b-5p	-1.27	0.42
112	mmu-miR-7029-5p	-1.25	0.42
113	mmu-miR-6912-3p	-1.24	0.42
114	mmu-miR-7659-5p	-1.21	0.43
115	mmu-miR-7664-3p	-1.21	0.43
116	mmu-miR-148a-5p	-1.20	0.43
117	mmu-miR-34b-5p	-1.18	0.44
118	mmu-miR-6948-5p	-1.15	0.45
119	mmu-miR-141-3p	-1.13	0.46
120	mmu-miR-6918-5p	-1.11	0.46
121	mmu-miR-3093-5p	-1.10	0.47
122	mmu-miR-3084-3p	-1.06	0.48

**Table 4:** Upregulated microRNAs in the RE group RE group: resistance exercise group

Rank	microRNAs	Log_2_ fold change	Ratio
1	mmu-miR-590-5p	5.69	51.56
2	mmu-miR-185-5p	3.38	10.40
3	mmu-miR-101a-3p	2.91	7.51
4	mmu-miR-155-5p	2.84	7.17
5	mmu-miR-463-5p	2.84	7.15
6	mmu-miR-23b-5p	2.72	6.58
7	mmu-miR-96-5p	2.54	5.82
8	mmu-miR-29c-5p	2.39	5.23
9	mmu-miR-369-3p	2.33	5.04
10	mmu-miR-465d-3p	2.30	4.92
11	mmu-miR-1970	2.25	4.75
12	mmu-miR-669g	2.23	4.68
13	mmu-miR-7229-3p	2.21	4.61
14	mmu-miR-1941-3p	2.17	4.49
15	mmu-miR-148b-3p	2.15	4.43
16	mmu-miR-1957b	2.13	4.39
17	mmu-miR-1947-5p	2.10	4.29
18	mmu-miR-450a-5p	2.07	4.21
19	mmu-miR-539-5p	2.05	4.13
20	mmu-miR-135b-3p	2.03	4.09
21	mmu-miR-7093-5p	2.03	4.08
22	mmu-miR-743a-3p	1.99	3.96
23	mmu-miR-7220-3p	1.98	3.93
24	mmu-miR-540-5p	1.97	3.93
25	mmu-miR-298-3p	1.96	3.90
26	mmu-miR-138-2-3p	1.92	3.79
27	mmu-miR-3962	1.89	3.70
28	mmu-miR-344-3p	1.89	3.70
29	mmu-miR-6984-3p	1.86	3.62
30	mmu-miR-5615-5p	1.83	3.56
31	mmu-miR-7649-3p	1.79	3.45
32	mmu-miR-466d-5p	1.77	3.41
33	mmu-miR-3105-3p	1.72	3.29
34	mmu-miR-509-5p	1.71	3.28
35	mmu-miR-1193-3p	1.70	3.25
36	mmu-miR-6238	1.64	3.11
37	mmu-miR-93-3p	1.64	3.11
38	mmu-miR-98-5p	1.62	3.07
39	mmu-miR-203-3p	1.51	2.85
40	mmu-let-7c-1-3p	1.44	2.72
41	mmu-miR-16-5p	1.29	2.44
42	mmu-miR-429-3p	1.27	2.42
43	mmu-miR-205-5p	1.27	2.41
44	mmu-miR-1933-3p	1.25	2.38
45	mmu-miR-33-3p	1.23	2.35
46	mmu-miR-7678-5p	1.23	2.35
47	mmu-miR-6989-5p	1.13	2.19
48	mmu-miR-144-3p	1.13	2.19
49	mmu-miR-126a-5p	1.12	2.17
50	mmu-miR-15b-5p	1.09	2.12
51	mmu-miR-695	1.08	2.11
52	mmu-miR-26b-5p	1.04	2.05
53	mmu-let-7a-5p	1.04	2.05
54	mmu-miR-8097	1.03	2.04
55	mmu-miR-195a-5p	1.03	2.04
56	mmu-miR-7094-1-5p	1.02	2.03

**Table 5:** Downregulated microRNAs in the RE group RE group: resistance exercise group

Rank	microRNAs	Log_2_ fold change	Ratio
1	mmu-miR-1901	-5.53	0.02
2	mmu-miR-6920-5p	-3.52	0.09
3	mmu-miR-7006-3p	-2.66	0.16
4	mmu-miR-802-3p	-2.52	0.17
5	mmu-miR-743b-5p	-2.51	0.18
6	mmu-miR-1843a-3p	-2.47	0.18
7	mmu-miR-6380	-2.33	0.20
8	mmu-miR-339-3p	-2.28	0.21
9	mmu-miR-302d-5p	-2.26	0.21
10	mmu-miR-6996-3p	-2.21	0.22
11	mmu-miR-7026-3p	-2.19	0.22
12	mmu-miR-7660-5p	-2.19	0.22
13	mmu-miR-7064-3p	-2.17	0.22
14	mmu-miR-467a-5p	-2.16	0.22
15	mmu-miR-329-5p	-2.15	0.22
16	mmu-let-7g-3p	-2.15	0.23
17	mmu-miR-669a-3-3p	-2.12	0.23
18	mmu-miR-654-3p	-2.09	0.24
19	mmu-miR-466o-3p	-2.07	0.24
20	mmu-miR-701-5p	-2.06	0.24
21	mmu-miR-1948-3p	-2.05	0.24
22	mmu-miR-370-5p	-2.05	0.24
23	mmu-miR-7216-3p	-2.03	0.24
24	mmu-miR-3063-3p	-2.02	0.25
25	mmu-miR-6933-3p	-2.01	0.25
26	mmu-miR-433-3p	-2.00	0.25
27	mmu-miR-1298-3p	-1.97	0.25
28	mmu-miR-302c-5p	-1.97	0.25
29	mmu-miR-691	-1.97	0.26
30	mmu-miR-7676-5p	-1.97	0.26
31	mmu-miR-202-3p	-1.96	0.26
32	mmu-miR-6417	-1.96	0.26
33	mmu-miR-6912-5p	-1.96	0.26
34	mmu-miR-1968-5p	-1.94	0.26
35	mmu-miR-6946-5p	-1.93	0.26
36	mmu-miR-382-5p	-1.93	0.26
37	mmu-miR-1668	-1.91	0.27
38	mmu-miR-6932-5p	-1.91	0.27
39	mmu-miR-3544-5p	-1.90	0.27
40	mmu-miR-1903	-1.88	0.27
41	mmu-miR-201-3p	-1.87	0.27
42	mmu-miR-3092-5p	-1.86	0.28
43	mmu-miR-708-5p	-1.86	0.28
44	mmu-miR-3106-5p	-1.86	0.28
45	mmu-miR-3967	-1.85	0.28
46	mmu-miR-7077-3p	-1.85	0.28
47	mmu-miR-293-3p	-1.83	0.28
48	mmu-miR-7688-5p	-1.82	0.28
49	mmu-miR-132-3p	-1.81	0.28
50	mmu-miR-495-5p	-1.80	0.29
51	mmu-miR-196a-1-3p	-1.79	0.29
52	mmu-miR-6957-3p	-1.79	0.29
53	mmu-miR-490-5p	-1.75	0.30
54	mmu-miR-3087-3p	-1.71	0.31
55	mmu-miR-6958-3p	-1.71	0.31
56	mmu-miR-7116-3p	-1.70	0.31
57	mmu-miR-6949-5p	-1.69	0.31
58	mmu-miR-7092-3p	-1.68	0.31
59	mmu-let-7e-3p	-1.67	0.31
60	mmu-miR-713	-1.66	0.32
61	mmu-miR-100-3p	-1.63	0.32
62	mmu-miR-1957a	-1.63	0.32
63	mmu-miR-5626-3p	-1.61	0.33
64	mmu-miR-92a-1-5p	-1.60	0.33
65	mmu-miR-3094-3p	-1.58	0.34
66	mmu-miR-411-5p	-1.57	0.34
67	mmu-miR-98-3p	-1.55	0.34
68	mmu-miR-871-3p	-1.54	0.34
69	mmu-miR-6407	-1.54	0.34
70	mmu-miR-6969-3p	-1.47	0.36
71	mmu-miR-217-5p	-1.45	0.37
72	mmu-miR-6374	-1.44	0.37
73	mmu-miR-7086-3p	-1.44	0.37
74	mmu-miR-669b-5p	-1.40	0.38
75	mmu-miR-5125	-1.39	0.38
76	mmu-miR-707	-1.38	0.38
77	mmu-miR-377-5p	-1.38	0.38
78	mmu-miR-1897-3p	-1.38	0.38
79	mmu-miR-3080-5p	-1.36	0.39
80	mmu-miR-325-3p	-1.36	0.39
81	mmu-miR-3062-5p	-1.35	0.39
82	mmu-miR-7657-3p	-1.35	0.39
83	mmu-miR-7675-3p	-1.33	0.40
84	mmu-miR-1912-5p	-1.33	0.40
85	mmu-miR-7660-3p	-1.32	0.40
86	mmu-miR-7a-1-3p	-1.31	0.40
87	mmu-miR-6898-3p	-1.31	0.40
88	mmu-miR-541-5p	-1.29	0.41
89	mmu-miR-7060-3p	-1.29	0.41
90	mmu-miR-7018-3p	-1.27	0.41
91	mmu-miR-467b-5p	-1.27	0.41
92	mmu-miR-141-3p	-1.26	0.42
93	mmu-miR-491-3p	-1.26	0.42
94	mmu-miR-7091-3p	-1.26	0.42
95	mmu-miR-7029-5p	-1.26	0.42
96	mmu-miR-7223-5p	-1.23	0.43
97	mmu-miR-7659-5p	-1.22	0.43
98	mmu-miR-148a-5p	-1.21	0.43
99	mmu-miR-34c-5p	-1.20	0.43
100	mmu-miR-34b-5p	-1.19	0.44
101	mmu-miR-6948-5p	-1.16	0.45
102	mmu-miR-6996-5p	-1.13	0.46
103	mmu-miR-6990-3p	-1.08	0.47
104	mmu-miR-133b-3p	-1.08	0.47
105	mmu-miR-669e-5p	-1.08	0.47
106	mmu-miR-692	-1.05	0.48
107	mmu-miR-3105-5p	-1.03	0.49
108	mmu-miR-5129-5p	-1.02	0.49
109	mmu-miR-1933-5p	-1.01	0.49
110	mmu-miR-344d-2-5p	-1.01	0.50
111	mmu-miR-6918-5p	-1.00	0.50

Target genes of the top 20 upregulated and downregulated microRNAs were predicted using miRWalk. As a result, 2426 to 2918 genes were detected. In the AE group, KEGG pathway analysis revealed that the pathway of proteoglycans in cancer was the highest ranked, followed by the mammalian target of rapamycin (mTOR) signaling pathway, and axon guidance. Of these pathways, mTOR signaling pathway, FoxO signaling pathway, transforming growth factor-β signaling pathway, phosphoinositide 3-kinase-Akt (PI3K-Akt) signaling pathway, 5'‐adenosine monophosphate-activated protein kinase (AMPK) signaling pathway, and mitogen-activated protein kinase (MAPK) signaling pathway were detected as the pathways related to molecular responses in skeletal muscle (Figure 2E). In the RE group, KEGG pathway analysis revealed that the pathway in cancer was the highest ranked, followed by the FoxO signaling pathway and Ras signaling pathway. FoxO signaling pathway, MAPK signaling pathway, insulin signaling pathway, insulin resistance pathway, and PI3K-Akt signaling pathway were detected as the pathways related to molecular responses in skeletal muscle (Figure 2F).

From altered microRNAs in microarray analysis, we selected miR-29c, miR-23b, miR-222, miR-195a, and miR-126 as candidates for biomarkers to determine moderate exercise intensity since these five microRNAs have been associated with molecular response in skeletal muscle [8-12] and expression of these five microRNAs have been reported to be altered after exercise [7,13-16]. In addition to these five microRNAs, miR-133a and miR-486 were also selected as these microRNAs have been also reported to be associated with exercise-induced molecular response [17,18]. Hence, miR-29c, miR-23b, miiR-222, miR-195a, miR-126, miR-133a, and miR-486, were analyzed for further analysis.

The target genes of these microRNAs are described below. Targets of miR-29c are collagen type IV α1 chain (COL4A) and COL4A2 via the PI3K/Akt signaling pathway [11] and it is involved in endothelial cell proliferation and vascularization. Targets of miR-23b and miR-486 are phosphatase and tensin homolog (PTEN) [8,19] and they are involved in muscle atrophy. The Target of miR-222 is p27 and it contributes to the modulation of cell proliferation [9]. The Target of miR-195 is the high-mobility group AT-hook 1 (Hmga1) and it is involved in muscle growth and cell proliferation [12]. miR-126 is a critical regulator of muscle growth and targets Abce1, Ccl2, Foxo33, and Vegfa [10]. miR-133a enhances myoblast proliferation via targeting serum response factor [17].

 Participant characteristics in aerobic exercise

 The characteristics and VO_2max_ of the participants are shown in Table 6. There were no baseline differences between groups.

**Table 6:** Participants characteristics in aerobic exercise study BMI: body mass index; HI group: high-intensity exercise group; LI group: low-intensity exercise group; SD: standard deviation

Variables	LI group, n=6, mean (SD)	HI group, n=6, mean (SD)	P-value
Age, years	26.33 (3.72)	24.33 (2.25)	0.287
Height, cm	170.58 (4.94)	170.67 (4.18)	0.975
Body weight, kg	68.40 (12.14)	61.82 (3.80)	0.234
BMI, kg/m^2^	23.50 (3.98)	21.25 (1.55)	0.225
VO_2max_, ml/min/kg	32.67 (7.36)	32.84 (5.35)	0.964

Changes in VO_2max_

**Figure 3:**
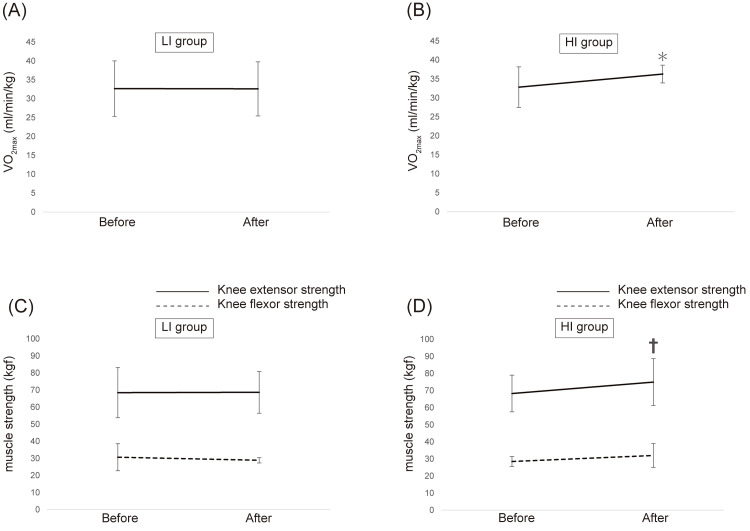
Changes in physical performance after four weeks of training period ^*^P<0.10: significant difference compared with the VO_2max_ before exercise. ^†^*P*<0.05: significant difference compared with the muscle strength before exercise (A-B) Changes in VO_2max_ after four weeks of aerobic exercise in (A) the LI group and (B) the HI group. (C-D) Changes in knee extensor (solid line) and knee flexor (dotted line) strength after four weeks of resistance exercise in (C) the LI group and (D) the HI group HI group: high-intensity exercise group; LI group: low-intensity exercise group

Figure 3A and Figure 3B show the changes in VO_2max_ after four weeks of aerobic exercise. In the HI group, VO_2max_ tended to improve (*p*=0.08), whereas no changes were noted in the LI group.

Circulating microRNAs in response to acute aerobic exercise

**Figure 4:**
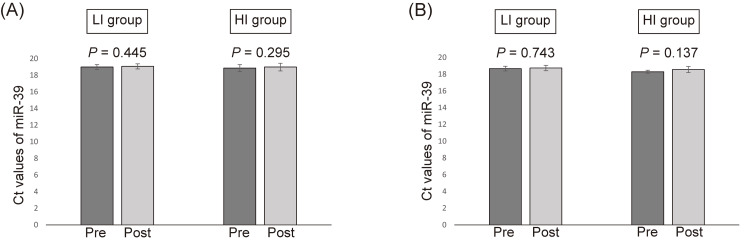
The Ct values of miR-39 The Ct values of miR-39 in plasma sample for LI and HI groups in the aerobic exercise study (A) and resistance exercise study (B). The total amount of miR-39 was measured in the same reverse transcription-quantitative polymerase chain reaction Ct: cycle threshold; HI group: high-intensity exercise group; LI group: low-intensity exercise group

For both groups, the Ct value for miR-39 before and after aerobic exercise showed low variability (Figure 4A).

**Figure 5:**
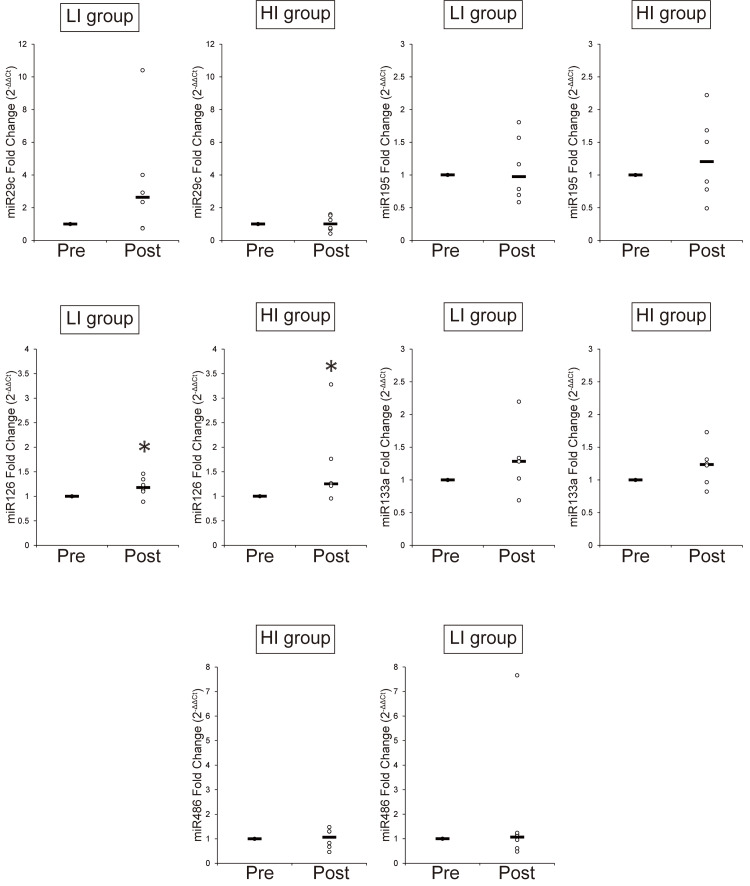
Changes in circulating microRNAs after four weeks of aerobic exercise ^*^P<0.10: significant difference compared with the value of Pre Circulating microRNAs before (Pre) and after (Post) four weeks of aerobic exercise normalized by miR-39 HI group: high-intensity exercise group; LI group: low-intensity exercise group

Figure 5 shows the changes in microRNA expressions in both groups. In both groups, miR-29c, miR-195, miR-126, miR-133a, and miR-486 remained unchanged, whereas miR-126 tended to be increased in both groups (*p*<0.10). The mean Ct value of miR-23b was 35.12, and that of miR-222 was 36.79. Moreover, Ct values of miR-23b and miR-222 were not detected in some samples. The cut-off value of low-copy (<250 copies) circulating microRNAs is typically 35 PCR cycles; below this value, microRNAs are difficult to quantify and accurately compare [20]. Therefore, we judged that miR-23b and miR-222 were difficult to compare regarding changes in response to exercise in human experiments.

Correlation between microRNAs and VO_2max_ changes after aerobic exercise

**Figure 6:**
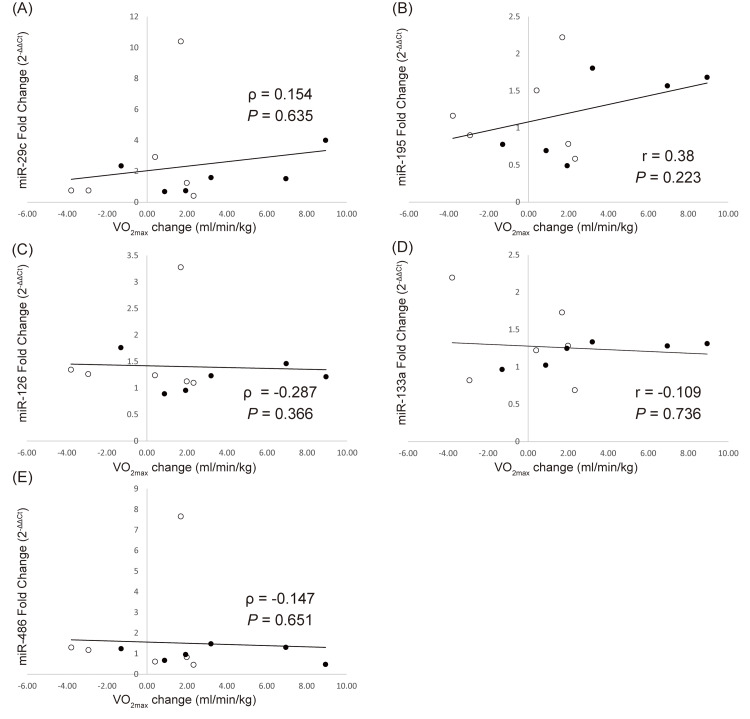
Linear correlations between VO2max changes and fold change of microRNAs Linear correlations between VO_2max_ changes after a four-week aerobic exercise period and fold change of (A) miR-29c, (B) miR-195, (C) miR-126, (D) miR-133a, and (E) miR-486 in response to acute aerobic exercise. Data are from Pearson’s correlation or Spearman’s rank correlation. The black dots represent data from the HI group and the white dots represent data from the LI group HI group: high-intensity exercise group; LI group: low-intensity exercise group

Figure 6 shows the correlation between microRNA expressions and changes in VO_2max_. No significant correlation was found between them.

Participant characteristics in resistance exercise

The characteristics and muscle strength of the participants are shown in Table 7. There were no baseline differences between the groups.

**Table 7:** Participants characteristics in resistance exercise study BMI: body mass index; HI group: high-intensity exercise group; IQR, interquartile range; LI group: low-intensity exercise group; SD: standard deviation

Variables	LI group, n=6	HI group, n=6	P-value
Age, years, mean (SD)	26.17 (4.40)	25.00 (3.22)	0.612
Height, cm, mean (SD)	170.33 (3.88)	171.50 (3.27)	0.586
Body weight, kg, median (IQR)	59.20 (58.70, 62.25)	62.30 (60.58, 67.10)	0.423
BMI, kg/m^2^, mean (SD)	22.07 (3.87)	21.77 (1.68)	0.862
Knee extensor strength, kgf, mean (SD)	68.51 (14.68)	68.29 (10.77)	0.977
Knee flexor strength, kgf, mean (SD)	30.71 (7.92)	28.50 (2.89)	0.535

Changes in muscle strength

Figure 3C and Figure 3D show the changes in muscle strength after four weeks of resistance exercise. In the HI group, knee extensor strength improved (*p*=0.02), whereas no changes were noted in the LI group. Knee flexor strength also improved in the HI group compared with the LI group, but without a statistically significant difference.

Circulating microRNAs in response to acute resistance exercise

**Figure 7:**
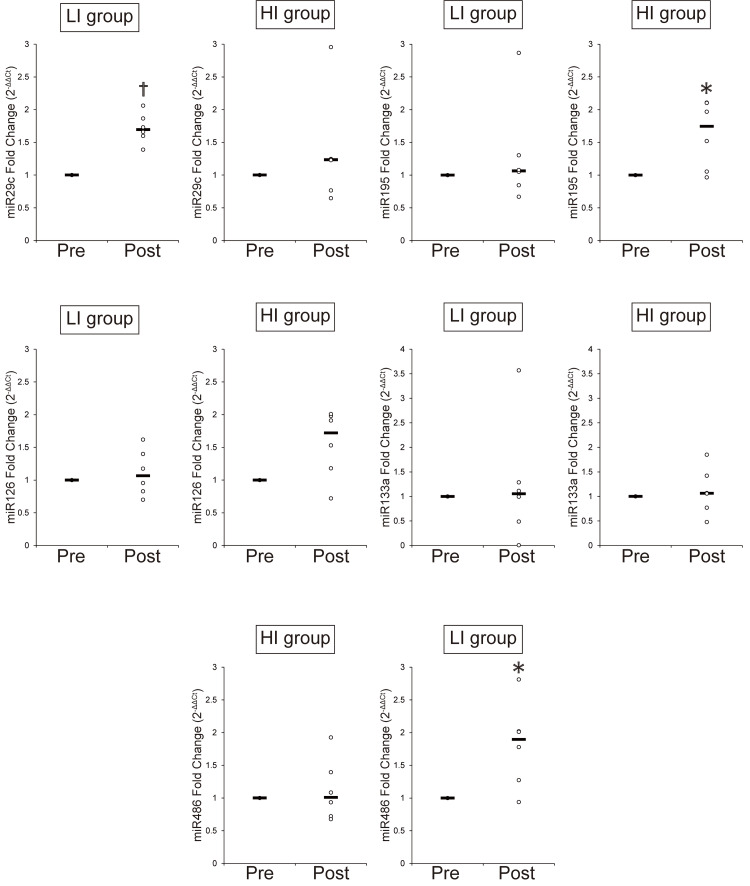
Changes in circulating microRNAs after four weeks of resistance exercise ^*^P<0.05: significant difference compared with the value of Pre. ^†^*P*<0.01: significant difference compared with the value of Pre Circulating microRNAs before (Pre) and after (Post) four weeks of resistance exercise normalized by miR-39 HI group: high-intensity exercise group; LI group: low-intensity exercise group

For both groups, the Ct value for miR-39 before and after resistance exercise showed low variability (Figure 4B). Figure 7 shows the changes in microRNA expressions in both groups. In the HI group, the levels of miR-29c, miR-195, and miR-486 significantly increased immediately after resistance exercise. Although no statistically significant difference was found, the levels of miR-126 tended to increase immediately after resistance exercise in the HI group (*p*=0.06). On the other hand, all microRNAs were unchanged in the LI group. The Ct values of miR-23b and miR-222 were not detected as well as in the aerobic exercise study. Therefore, we excluded miR-23b and miR-222 from further analysis.

Correlation between microRNAs and muscle strength changes after resistance exercise

**Figure 8:**
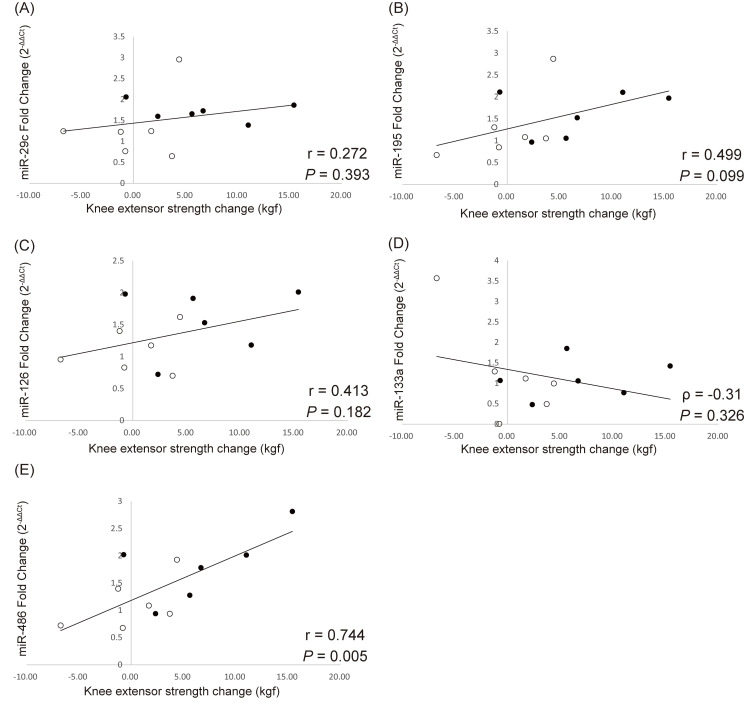
Linear correlations between muscle strength changes and fold change of microRNAs Linear correlations between muscle strength changes after a four-week resistance exercise period and fold change of (A) miR-29c, (B) miR-195, (C) miR-126, (D) miR-133a, and (E) miR-486 in response to the acute resistance exercise. Data are from Pearson’s correlation or Spearman’s rank correlation. The black dots represent data from the HI group and the white dots represent data from the LI group HI group: high-intensity exercise group; LI group: low-intensity exercise group

Figure 8 shows the correlation between microRNAs and changes in muscle strength. Of the five microRNAs, the fold change of miR-486 significantly correlated with the changes in knee extensor strength (r=0.744, *p*=0.005). No other statistically significant correlations were observed between muscle strength and other microRNAs.

## Discussion

In this study, we comprehensively explored the alterations in circulating microRNAs after exercise at the effective intensity in mice. Combining our findings with some published reports, we identified miR-29c, miR-23b, miR-222, miR-195, miR-126, miR-133a, and miR-486 as the candidates for objective indicators to determine effective exercise intensity. In our human study, post-exercise expressions of miR-29c, miR-195, and miR-486 were different in resistance exercise depending on exercise intensity, although aerobic exercise had no effect on post-exercise expression of these microRNAs. The important insights we gained were that these three microRNAs did not alter after low-intensity exercise which had no effect on muscle strength, whereas the expressions of these microRNAs were upregulated by high-intensity exercise which improved muscle strength. Therefore, these microRNAs may serve as objective indicators to determine the intensity of resistance exercise.

One of the strengths of our study is the method of exploring seven microRNAs that were candidates for biomarkers to determine the effective exercise intensity. Many published studies have only explored microRNAs that were altered after exercise, and whether these microRNAs were altered by moderate exercise was unclear. Medium- to vigorous-intensity exercises have been recommended in humans [2,3]; therefore, we exercised mice at medium-to-vigorous intensity and explored circulating microRNAs comprehensively. As moderate intensity is variable among individuals and difficult to identify, comprehensive exploration in genetically homogeneous laboratory animals must provide more accurate results than on humans. We believe our methods of exploring microRNAs are uniform and novel.

Targets of miR-29c are COL4A and COL4A2 via PI3K/Akt signaling pathway and are involved in endothelial cell proliferation and vascularization in skeletal muscle [11]. Previous studies [13] have shown the upregulation of miR-29c after high-intensity interval training. Although some studies have described the differential expression of miR-29c after aerobic exercise, to the best of our knowledge, no studies have reported the differential expression of miR-29c after resistance exercise. However, given that resistance exercise as well as aerobic exercise promotes vascularization [21], the differential expression of miR-29c after resistance exercise can be explained. The target of miR-195 is Hmga1, which is involved in muscle growth and cell proliferation [12].

In Vogel et al.’s study [7], miR-195 was not altered by low-intensity resistance exercise, whereas high-intensity resistance exercise upregulated the expression of miR-195; our results are in line with this study [7]. Another published study [22] has reported the upregulation of circulating miR-195 after resistance exercise, also in agreement with our results. Target of miR-486 is PTEN [19], and miR-486 deficient mice had decreased muscle mass and muscle cross-sectional area [18]. Increased miR-486 in skeletal muscle led to phosphorylation of Akt and FOXO1, inhibition of MAFbx/Atrogin-1, MuRF-1, and myostatin, and consequently contributed to the attenuation of muscle atrophy [23].

Another important insight into microRNA expression after resistance exercise was that fold changes of miR-486 significantly correlated with the changes in muscle strength. Hagstrom et al. [24] reported a positive correlation between fold change of miR-486 after 16 weeks of resistance exercise and lower body strength change. Our results partially support their study's findings. Although their study showed a correlation between long-term changes in miR-486 and muscle strength changes, our results showed a correlation between acute changes in miR-486 and muscle strength changes. Therefore, monitoring acute changes in miR-486 may serve as a means of predicting the resistance exercise response. Evaluation of circulating miR-486 immediately after resistance exercise may aid in determining the effective exercise intensity.

Resistance exercise altered microRNA expression, whereas aerobic exercise did not. In this study, four weeks of high-intensity aerobic exercise had no effect on VO_2max_. Seven microRNAs were selected as candidates for objective indicators to determine effective exercise intensity. Exercise workload used as a high intensity (70-80% of VO_2max_) may not be moderate for improving VO_2max_ and lead to unchanged microRNA expression. However, aerobic exercise altered circulating microRNAs [13,16], and our findings conflict with these studies. On the other hand, a previous study [25] reported unchanged microRNA expression immediately after exercise. Furthermore, even in the same microRNA, some studies reported the upregulation [26] and others reported the downregulation [27]. MicroRNA expression after exercise is contradictory, which could be attributed to the fact that no reference genes for microRNA quantification have been developed [28]. Although miR-39 as the external spike-in control was used commonly, the lack of developed reference genes may contribute to the inconsistency between our results and published studies.

Our study has several limitations. Firstly, the sample size was small in a human study. Although the sample size was determined based on similar human studies [29], future studies with larger numbers of participants are needed. A second limitation is that we formed only low- and high-intensity groups. We needed to detect obvious differences in the effects of exercise between low- and high-intensity exercise. Resistance exercises with higher intensity provide muscle hypertrophy and muscle mass gain [3]. High-intensity aerobic exercises are also recommended [2]. Based on these reports, we set 80% 1RM for resistance exercise and 70-80%VO_2max_ for aerobic exercise as a high-intensity exercise. In further studies, exercise intensity should be subclassified and reproducibility needs to be evaluated.

A third limitation is that we recruited only healthy males. There were sex differences in microRNA expression in skeletal muscle after exercise [30]. In addition, expressions of microRNAs are affected by various diseases. We recruited only healthy males to adjust for confounders such as sex and disease. Whether our results can be generalized to females or patients with diseases is still controversial. Further studies are needed to expand the subject population. We can warrant external validity by analyzing whether microRNAs are altered by exercise independently of gender and disease. A final limitation is the use of miR-39 as a reference gene. As miR-39, a spike-in control, was added during RNA extraction, it may be degraded. On the other hand, the reference genes for internal control include miR-191 and miR-16. However, miR-16 was upregulated after resistance exercise in mice (Table 4) and miR-191 may also be altered after exercise. In this study, the Ct value for miR-39 before and after resistance exercise showed low variability (Figure 4), and the total amount of miR-39 was measured in the same reverse transcription-quantitative polymerase chain reaction. Therefore, we believe that the use of miR-39 as a reference gene is appropriate.

## Conclusions

Our findings showed that resistance exercise at the effective intensity upregulated the expression of miR-29c, miR-195, and miR-486, although aerobic exercise had no effect on the seven microRNAs selected as candidates of objective indicators to determine moderate exercise intensity. These three microRNAs may serve as objective indicators to determine the intensity of resistance exercise. Among them, fold change of miR-486 correlated with changes in muscle strength, and therefore, miR-486 may aid in predicting the response to resistance exercise.
